# Supplementary material for: Evaluating the effectiveness of a population-level health intervention to increment HCV treatment coverage in tuscany region, Italy: An interrupted time series analysis
Source: PLoS One. 2025 May 16;20(5):e0306733. doi: 10.1371/journal.pone.0306733 (PMC12084055; doi:10.1371/journal.pone.0306733)
Supplement: S1 Table — Coefficients are shown for five groups: General, General (33–55 y.o), PWUD (People Who Use Drugs), PLP (Precariously Living Persons), and PWUD + PLP. Variables include an intervention indicator (interven), COVID-19 period (covid), lagged serological tests (n_serological_lagged2), and time. ARIMA terms: ar1, ar2 (autoregressive), and ma1 (moving average). Standard errors in parentheses; p < 0.05, p < 0.01, p < 0.001. Model fit: log-likelihood (logLik), Akaike (AIC), and Bayesian (BIC) criteria. (DOCX) [file pone.0306733.s001.docx]

|  | General | General, 33 - 55 y.o | PWUD | PLP | PWUD + PLP |
| --- | --- | --- | --- | --- | --- |
| interven | 1.16 * | 1.19 * | 0.21 | 0.77 | 0.43 |
|  | (0.54) | (0.60) | (0.54) | (0.51) | (0.55) |
| covid | -1.51 *** | -1.49 ** | -1.22 * | -0.73 | -1.23 * |
|  | (0.45) | (0.48) | (0.52) | (0.47) | (0.53) |
| n_serological_lagged2 | -0.00 *** | -0.01 *** | -0.02 * | -0.04 * | -0.01 * |
|  | (0.00) | (0.00) | (0.01) | (0.02) | (0.01) |
| time | -0.01 | -0.01 | 0.02 | 0.01 | 0.01 |
|  | (0.01) | (0.01) | (0.01) | (0.01) | (0.01) |
| ar1 | -0.45 *** | -0.42 *** | 0.16 | 0.82 *** | 0.23 * |
|  | (0.11) | (0.11) | (0.10) | (0.12) | (0.11) |
| ar2 | 0.49 *** | 0.52 *** | 0.29 ** |  | 0.25 * |
|  | (0.10) | (0.10) | (0.10) |  | (0.10) |
| ma1 | 0.82 *** | 0.82 *** |  | -0.64 *** |  |
|  | (0.08) | (0.08) |  | (0.16) |  |
| nobs | 96 | 96 | 96 | 94 | 96 |
| logLik | -91.01 | -95.94 | -118.73 | -110.84 | -116.50 |
| AIC | 200.03 | 209.89 | 251.46 | 235.68 | 247.01 |
| BIC | 223.11 | 232.97 | 269.41 | 253.49 | 264.96 |
| nobs.1 | 96.00 | 96.00 | 96.00 | 94.00 | 96.00 |
| *** p < 0.001; ** p < 0.01; * p < 0.05. | | | | | |
