# Supplementary figures and images for: Evaluating the effectiveness of a population-level health intervention to increment HCV treatment coverage in tuscany region, Italy: An interrupted time series analysis
Source: PLoS One. 2025 May 16;20(5):e0306733. doi: 10.1371/journal.pone.0306733 (PMC12084055; doi:10.1371/journal.pone.0306733)

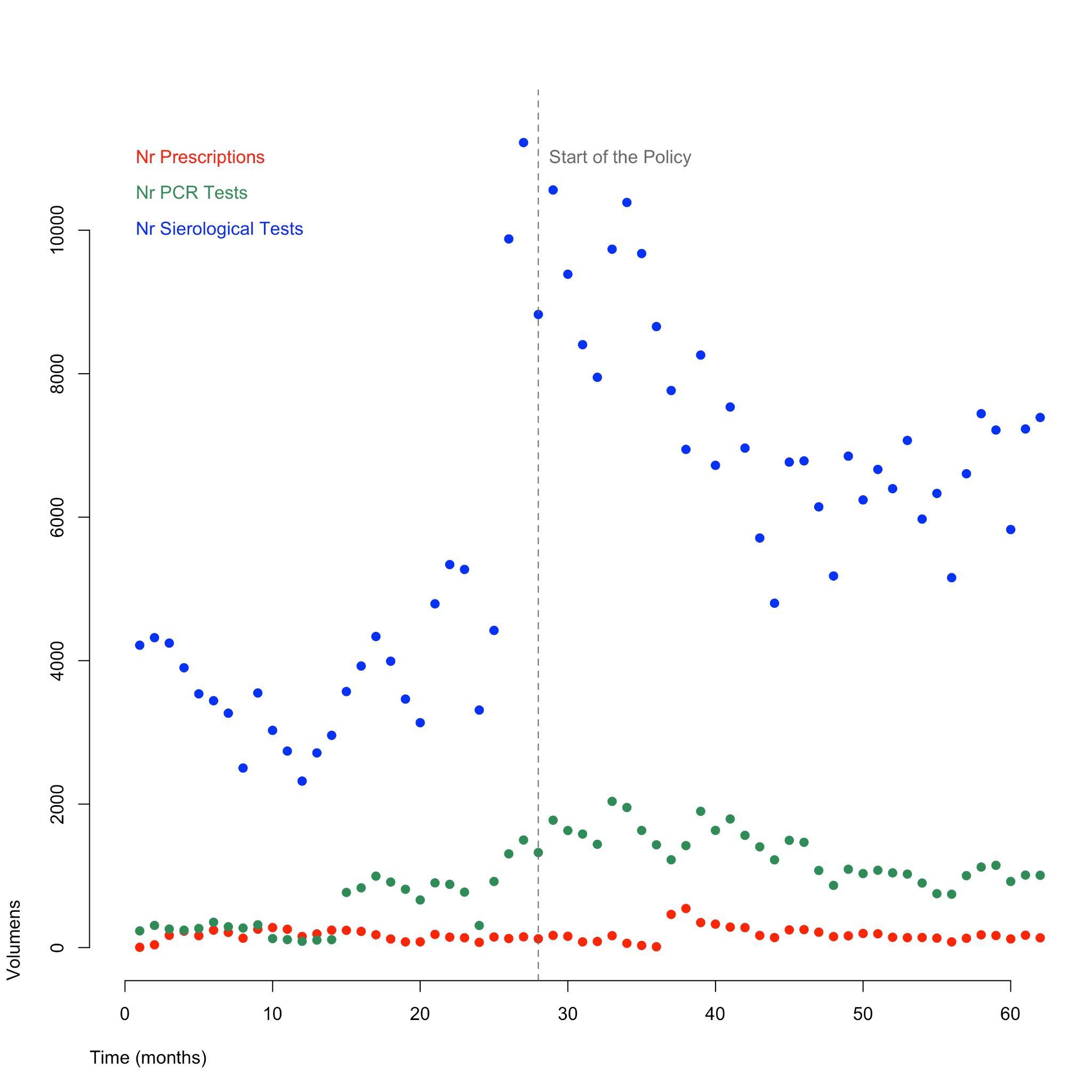

Supplement: S1 Fig — Monthly volumes of prescriptions (red), PCR tests (green), and serological tests (blue) are plotted over a 60-month period. The dashed vertical line marks the start of a policy intervention. A visible change in levels and trends, particularly in serological testing, suggests a potential policy impact. Y-axis represents volumes; X-axis represents time in months. (DOCX) [file pone.0306733.s002.docx]

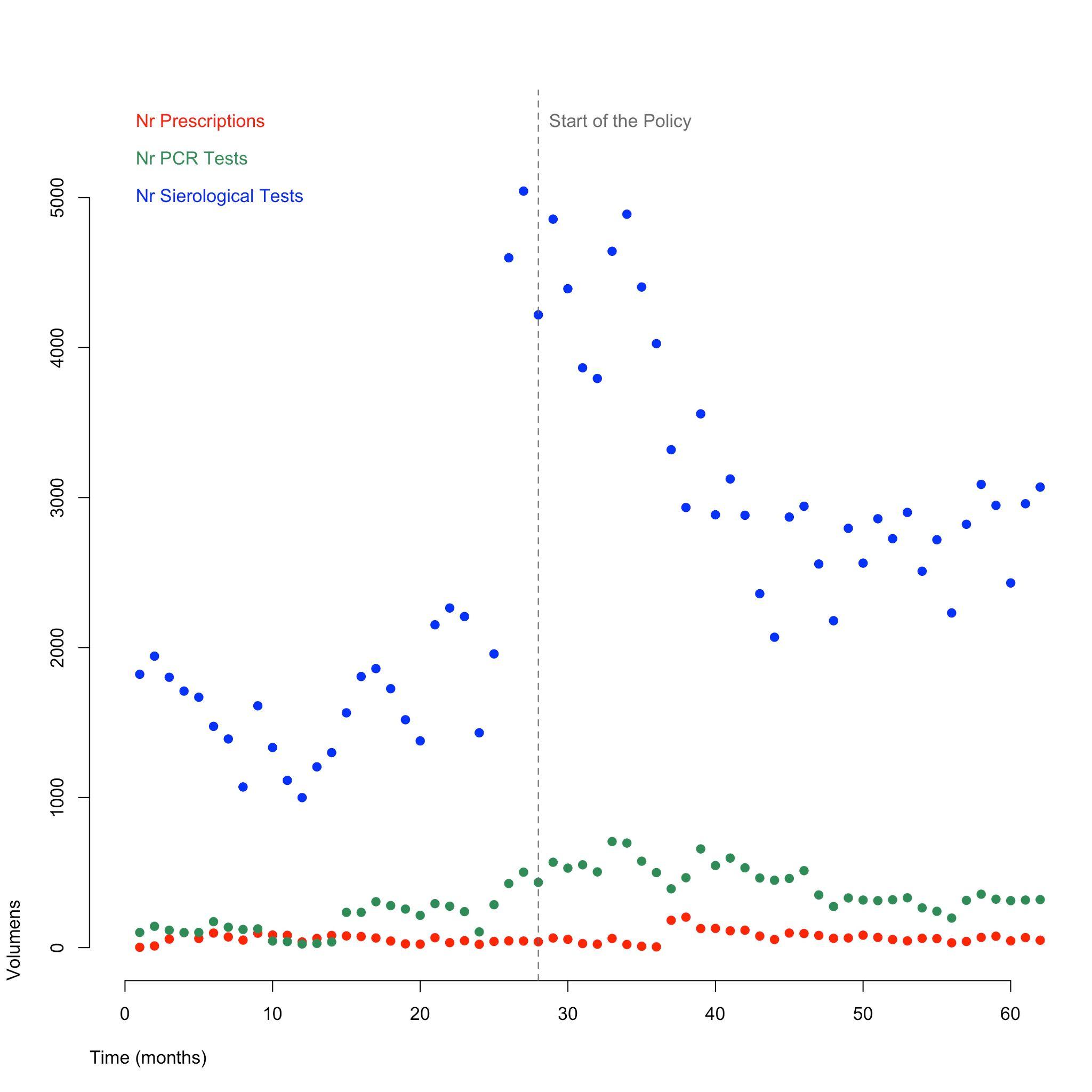

Supplement: S2 Fig — Monthly volumes of prescriptions (red), PCR tests (green), and serological tests (blue) are plotted over a 60-month period. The dashed vertical line marks the start of a policy intervention. A visible change in levels and trends, particularly in serological testing, suggests a potential policy impact. Y-axis represents volumes; X-axis represents time in months. (DOCX) [file pone.0306733.s003.docx]

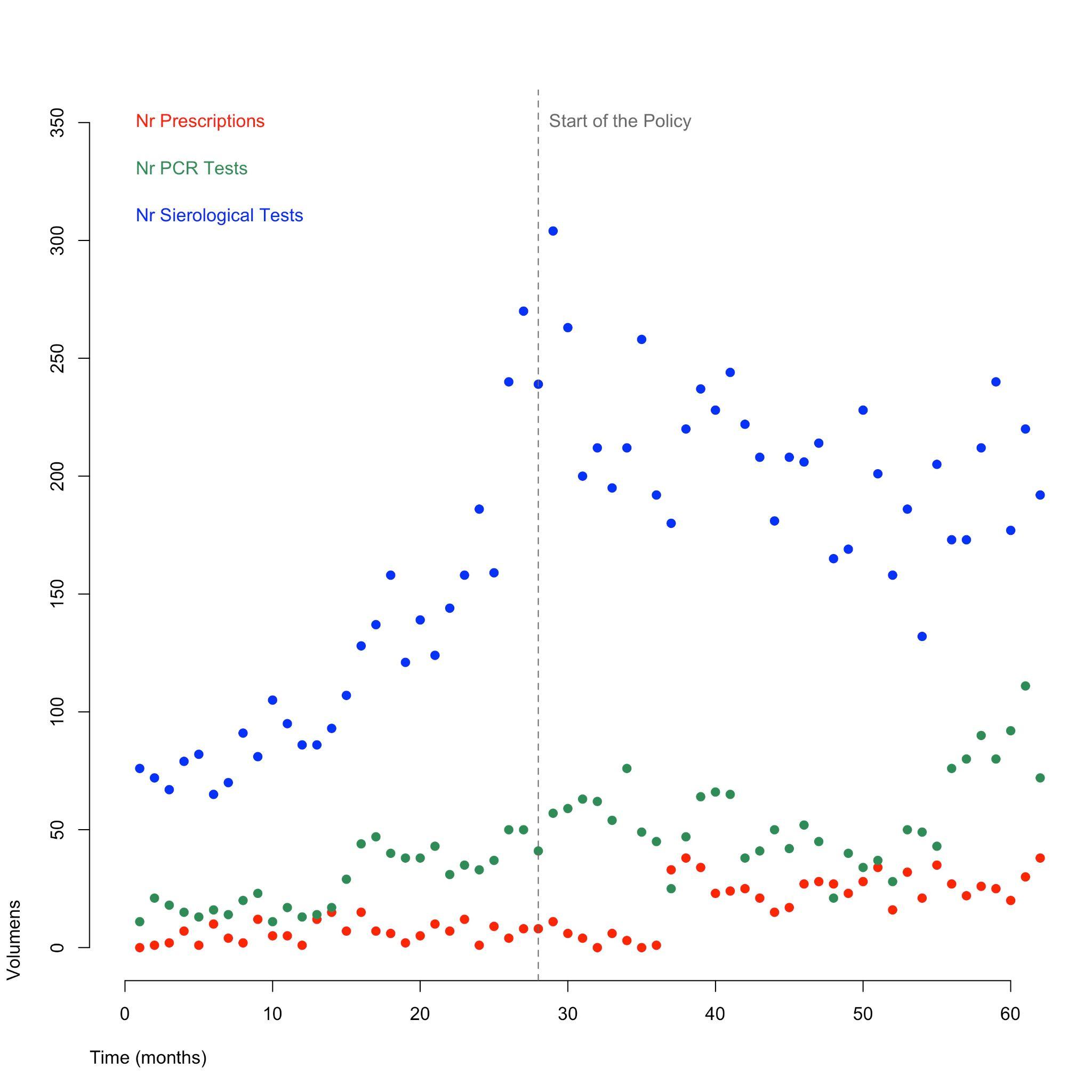

Supplement: S3 Fig — Monthly volumes of prescriptions (red), PCR tests (green), and serological tests (blue) are plotted over a 60-month period. The dashed vertical line marks the start of a policy intervention. A visible change in levels and trends, particularly in serological testing, suggests a potential policy impact. Y-axis represents volumes; X-axis represents time in months. PWUD: People Who Use Drugs; PLP: People Living in Prison. (DOCX) [file pone.0306733.s004.docx]

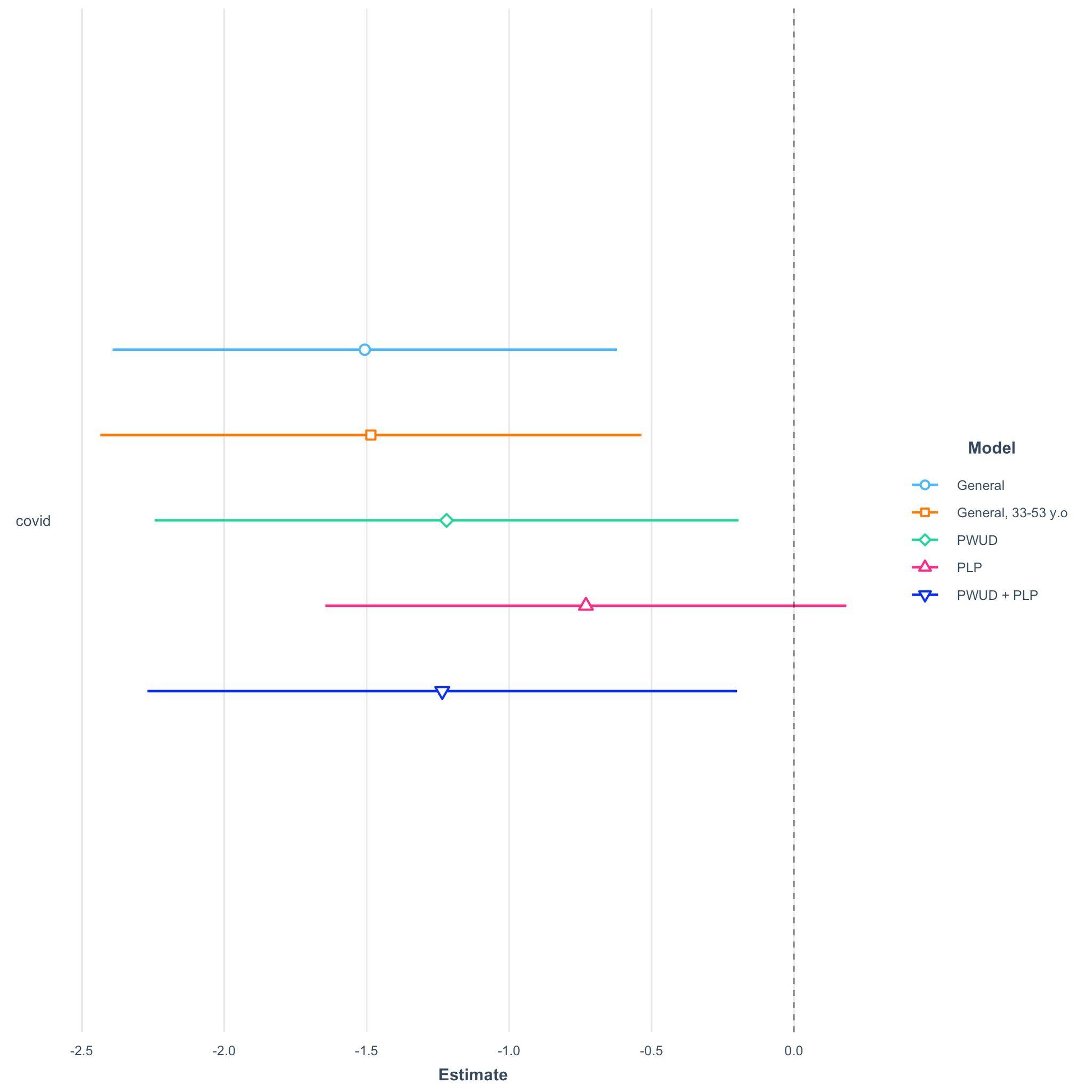

Supplement: S4 Fig — Point estimates and 95% confidence intervals for the covid variable are shown across five ARIMA models: General, General (33–55 y.o), PWUD (People Who Use Drugs), PLP (Precariously Living Persons), and PWUD + PLP. All estimates indicate a negative association between the COVID-19 period and the outcome variable. The vertical dashed line marks the null effect (estimate = 0). (DOCX) [file pone.0306733.s005.docx]
